# A phase II single‐arm trial of memantine for prevention of cognitive decline during chemotherapy in patients with early breast cancer: Feasibility, tolerability, acceptability, and preliminary effects

**DOI:** 10.1002/cam4.5619

**Published:** 2023-01-16

**Authors:** Zev M. Nakamura, Allison M. Deal, Eliza M. Park, Kate E. Stanton, Yesy E. Lopez, Laura J. Quillen, Erin O’Hare Kelly, Hillary M. Heiling, Kirsten A. Nyrop, Emily M. Ray, E. Claire Dees, Katherine E. Reeder‐Hayes, Trevor A. Jolly, Lisa A. Carey, Yara Abdou, Oludamilola A. Olajide, Julia K. Rauch, Ranjit Joseph, Anureet Copeland, Megan A. McNamara, Tim A. Ahles, Hyman B. Muss

**Affiliations:** ^1^ Department of Psychiatry University of North Carolina at Chapel Hill Chapel Hill North Carolina USA; ^2^ Lineberger Comprehensive Cancer Center University of North Carolina at Chapel Hill Chapel Hill North Carolina USA; ^3^ Division of Oncology, Department of Medicine University of North Carolina at Chapel Hill Chapel Hill North Carolina USA; ^4^ Rex Hematology Oncology Associates Rex Cancer Care Raleigh North Carolina USA; ^5^ Department of Psychiatry and Behavioral Sciences Memorial Sloan Kettering Cancer Center New York New York USA

**Keywords:** behavioral science, breast cancer, cancer‐related cognitive impairment, clinical trials, cognitive dysfunction, memantine, quality of life

## Abstract

**Background:**

Cognitive difficulties have been described after chemotherapy for breast cancer, but there is no standard of care to improve cognitive outcomes in these patients. This trial examined the feasibility, tolerability, acceptability, and preliminary effects of memantine to prevent cognitive decline during chemotherapy for breast cancer.

**Methods:**

Patients with stage I–III breast cancer, scheduled for neo/adjuvant chemotherapy, completed a cognitive battery prior to and 4 weeks after completing chemotherapy. Memantine (10 mg BID) was administered concurrent with chemotherapy. Our primary cognitive outcome was visual working memory assessed by the Delayed Matching to Sample test. We used the Brief Medication Questionnaire to assess acceptability.

**Results:**

Of 126 patients approached, 56 (44%) enrolled. Forty‐five (80%) received ≥1 dose of memantine and completed pre‐post assessments. Seventy‐six percent reported taking ≥90% of scheduled doses. Participants were mean age of 56, 77% White, and 57% had stage I disease. Sixty‐four percent had stable or improved Delayed Matching to Sample test scores. Stable or improved cognition was observed in 87%–91% across objective cognitive domain composite measures. Sixty‐six percent self‐reported stable or improved cognitive symptoms. There were seven greater than or equal to grade 3 adverse events; two were possibly related to memantine. Only 5% reported that taking memantine was a disruption to their lives.

**Conclusions:**

Memantine was well‐tolerated and consistently taken by a large majority of patients receiving breast cancer chemotherapy. The majority demonstrated stable or improved cognition from pre‐ to post‐assessment. Randomized trials are needed to determine memantine's efficacy to ameliorate cognitive loss.

**Trial Registration:**

ClinicalTrials.gov NCT04033419.

## Graphical abstract

Memantine is a feasible, safe, and acceptable intervention to mitigate cognitive decline in patients who receive chemotherapy for early‐stage breast cancer. The majority demonstrated stable or improved cognition from pre‐ to post‐assessment. Randomized trials are needed to determine memantine's efficacy to ameliorate cognitive loss.

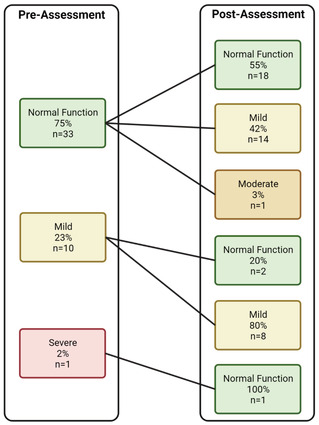

## 1 INTRODUCTION

Cancer‐related cognitive impairment (CRCI) is a common and consequential condition described in many cancers and following a myriad of cancer‐directed therapies.^1^, ^2^, ^3^ One suspected cause of CRCI is chemotherapy, with as many as 75% of patients with breast cancer self‐reporting cognitive decline during chemotherapy.^1^ CRCI can present major barriers to returning to pre‐cancer occupational and social role function and has been described as one of the most concerning issues for cancer survivors.^4^, ^5^

Pharmacotherapies for CRCI present potential advantages over behavioral interventions, including targeting precise mechanisms, minimizing patient time investment, and requiring few facility resources. However, there have been <10 randomized controlled trials (RCTs) of medications for CRCI and the only agents that have been studied for CRCI prevention during chemotherapy–Ginkgo biloba, erythropoietin, and methylphenidate–have not been effective and/or presented acceptability and safety concerns.^6^, ^7^ This gap is critical because: (1) CRCI is common; (2) associated with substantial morbidity; and (3) may represent irreversible changes to brain structure and function.^8^

Memantine, an *N*‐methyl‐d‐aspartate receptor antagonist, is a compelling medication to investigate for CRCI prevention during chemotherapy. Multi‐center studies in patients with brain metastases undergoing whole brain radiotherapy have demonstrated that memantine mitigates decline in executive function, memory, and processing speed and is now included in guideline recommendations.^9^, ^10^, ^11^ More recently, pre‐clinical studies suggest that memantine may prevent cognitive effects of chemotherapy through modulation of neuroinflammation and brain‐derived neurotrophic factor, ultimately preserving hippocampal neurogenesis.^12^, ^13^ Nonetheless, memantine's potential promise for CRCI has yet to be studied in clinical populations during chemotherapy. In this single‐arm trial, we assessed the feasibility, tolerability, and acceptability of memantine administration concurrent with neo/adjuvant chemotherapy for breast cancer. We examined pre‐post changes in objective and patient‐reported cognition.

## 2 METHODS

### 2.1 Design and study sample

In this phase II, single‐arm trial, we recruited 56 patients scheduled to begin neo/adjuvant chemotherapy for stage I–III breast cancer at the University of North Carolina (UNC) Health System's North Carolina Cancer Center, Hillsborough Hospital, and Rex Cancer Center between September 2019 and August 2021. Participants completed a cognitive assessment prior to initiating chemotherapy (pre‐) and 4 weeks after (post‐) their final cycle. Memantine was administered twice daily (BID) between pre‐ and post‐assessments. The study was approved by the UNC Institutional Review Board and UNC Lineberger Protocol Review Committee. The trial is registered at ClinicalTrials.gov number NCT04033419.

### 2.2 Eligibility criteria

Participants were: (1) diagnosed with stage I–III breast cancer; (2) scheduled for neo/adjuvant chemotherapy; (3) ≥18 years of age; (4) able to speak English; and (5) able to provide informed consent. Exclusion criteria were: (1) history of an adverse reaction to memantine; (2) another cancer diagnosis with estimated survival <5 years; (3) previous chemotherapy; (4) severe cognitive impairment, defined as Blessed Orientation‐Memory‐Concentration Test^14^ score ≥11; (5) pregnant or breast feeding; or (6) current alcohol or drug abuse.

### 2.3 Intervention

Participants started memantine no earlier than 4 weeks prior and no later than 1 week after their first cycle of chemotherapy. The dose was titrated as follows: 5 mg daily in week 1, 5 mg BID in week 2, 5 mg each morning, and 10 mg each evening in week 3, then 10 mg BID in week 4. Participants continued 10 mg BID (or highest tolerated dose) until 4 weeks after (range: 2–12 weeks) their final cycle of chemotherapy.

### 2.4 Acceptability

The Brief Medication Questionnaire (BMQ)‐Specific contains 10, 5‐point Likert scale items (1 = strongly disagree to 5 = strongly agree) that assess patients' beliefs about the necessity of and concerns about taking a medication for their illness.^15^ We modified items to reflect statements specific to memantine and cognition.

### 2.5 Adverse events (AE)

Participants were evaluated for toxicity with the National Cancer Institute Common Toxicity Criteria for Adverse Events (NCI CTCAE v 5) weekly during the 4‐week titration and every 2–3 weeks thereafter, coinciding with chemotherapy infusion. Though all patient‐reported symptoms were recorded, we explicitly solicited the most common side effects of memantine (headache, dizziness, confusion, constipation, diarrhea, and fatigue). Attribution was defined as *unrelated*, *unlikely*, *possible*, *probable*, or *definite* based on a priori criteria (Table S1). To provide additional indirect safety information, independent of AE attribution, we monitored the rate and reasons for chemotherapy dose reduction.

### 2.6 Measures

#### 2.6.1 Neuropsychological assessment

The Cambridge Neuropsychological Test Automated Battery (CANTAB) is comprised of highly sensitive and well‐validated computer‐based tests of cognition.^16^ Three CANTAB measures–Delayed Matching to Sample (DMS), Rapid Visual Processing, and One Touch Stockings of Cambridge–were chosen for this study based on their previous use in the CRCI field.^17^, ^18^, ^19^, ^20^ We chose the DMS % correctly at 12 s delay, a measure of visual working memory, as our primary outcome as it was recently shown to be especially sensitive to CRCI.^17^ During the DMS, participants are briefly shown a sample image with a complex visual pattern. Then, after a delay (up to 12 s long), they are asked to identify from a set of four similar appearing patterns the image that exactly matches the original sample.

Additionally, we administered paper‐based, traditional neuropsychological measures with well‐established track records in oncology (Table S2). To facilitate interpretability of our objective cognitive outcomes, we also constructed composite measures of attention, working memory, and executive function (AWE), learning and memory (LM), and global cognition, as has been described in prior CRCI studies (Table S2).^21^, ^22^ Though the original protocol was designed for in‐person assessment, at the start of the COVID‐19 pandemic, the protocol was modified to give participants the choice of whether to complete the cognitive assessment in‐person or remotely.

#### 2.6.2 Patient‐reported outcome measures

We used PROMIS measures to evaluate patient‐reported cognition (Short Form v2.0—Cognitive Function 8a), depression, anxiety, and sleep disturbance.^23^, ^24^, ^25^ Scores are reported as a *T*‐score with a mean of 50 and standard deviation (SD) of 10. Higher scores represent better cognitive function, but more severe depression, anxiety, and sleep disturbance. We utilized established thresholds to define normal cognitive function (>45) and mild (>35–45), moderate (>30–35), and severe (≤30) cognitive difficulties.^25^ For other PROMIS measures, we examined the proportion with at least moderately severe symptoms.^24^, ^25^

#### 2.6.3 Disease characteristics

Tumor stage, HER2 and hormone receptor (HR) positivity, history of surgery and radiation, and chemotherapy regimen were abstracted from the medical record.

### 2.7 Statistical analysis

#### 2.7.1 Power and sample size

For the primary cognitive outcome (change in DMS % correct at 12 s delay), we determined that a sample size of 45 participants was needed. A paired *t*‐test with 0.05 one‐sided significance level has 80% power to detect an effect size of 0.38 (3% mean increase in the DMS score with SD of 8%) compared to the null hypothesized change from a historical control of patients with early‐stage breast cancer receiving chemotherapy who did not receive any intervention for cognition.^17^ To account for dropout of up to 20%, we enrolled 56 participants.

#### 2.7.2 Data analysis

Descriptive statistics were used to characterize participants and evaluate aspects of feasibility (recruitment, retention, adherence), tolerability, and acceptability. A paired *t*‐test was used to test if the change in DMS score between pre‐post assessments was statistically different from historical controls. To evaluate other changes in objective cognition, we evaluated meaningful pre‐ to post‐assessment improvement or decline (defined as ≥0.5 SD) in all composite domains and individual measures. For patient‐reported cognition, we used established thresholds to define clinically meaningful changes (e.g., normal function to mild difficulties, mild difficulties to moderate difficulties, etc.).^25^ A priori we stipulated that all participants who received at least one dose of memantine would be evaluated for toxicity and that all participants who received at least one dose of memantine and completed the DMS at pre‐ and post‐timepoints would be evaluated for cognitive changes. We compared participant characteristics between the 45 subjects who completed the primary cognitive outcome and the 11 who did not using Fisher's exact tests for categorical and Wilcoxon rank sum tests for continuous characteristics.

## 3 RESULTS

### 3.1 Participant characteristics

On average, enrolled participants were 56 years old (range: 27–78; Table 1). Seventy‐seven percent were White, 18% were Black, and 5% were other races. Fifty‐seven percent had stage I disease, 68% had HR+ tumors, and 29% HER2+ tumors. Sixty‐four percent had prior lumpectomy or mastectomy. At least moderately severe psychological comorbidities were present in 6% (anxiety), 2% (depression), and 26% (sleep disturbance) at baseline and 11% (anxiety), 2% (depression), and 20% (sleep disturbance) at post‐assessment. Participants who initiated memantine and completed both pre‐ and post‐assessments had higher levels of education compared to those who did not (16.1 vs. 14.1 years, *p* = 0.03); otherwise, there were no significant differences between groups.

**TABLE 1:** Participant characteristics (*N* = 56)

	*N* (%)
Age, mean (SD), [range], years	56.2 (12.8), [27–78]
Female	55 (98.2)
Race	
White	43 (76.8)
Black	10 (17.9)
Other	3 (5.3)
Ethnicity	
Not Hispanic/Latino	54 (96.4)
Hispanic/Latino	2 (3.6)
Education (years), mean (SD)	15.8 (2.2)
Post‐menopausal	28 (59.6)
Stage	
I	32 (57.1)
II	15 (26.8)
III	9 (16.1)
HER2+	16 (28.6)
HR+	38 (67.9)
Chemotherapy timing	
Adjuvant	36 (64.3)
Neoadjuvant	20 (35.7)
Chemotherapy regimen	
Anthracycline‐based	24 (42.9)
Not anthracycline‐based	32 (57.1)
Prior radiation	2 (3.6)
Anxiety (PROMIS anxiety ≥65)	3 (5.9)
Depression (PROMIS depression ≥65)	1 (2.0)
Sleep disturbance (PROMIS sleep disturbance ≥55)	13 (26.0)

Abbreviations: HR, hormone receptor; PROMIS, patient‐reported outcomes measurement information system.

### 3.2 Feasibility

**FIGURE 1:**
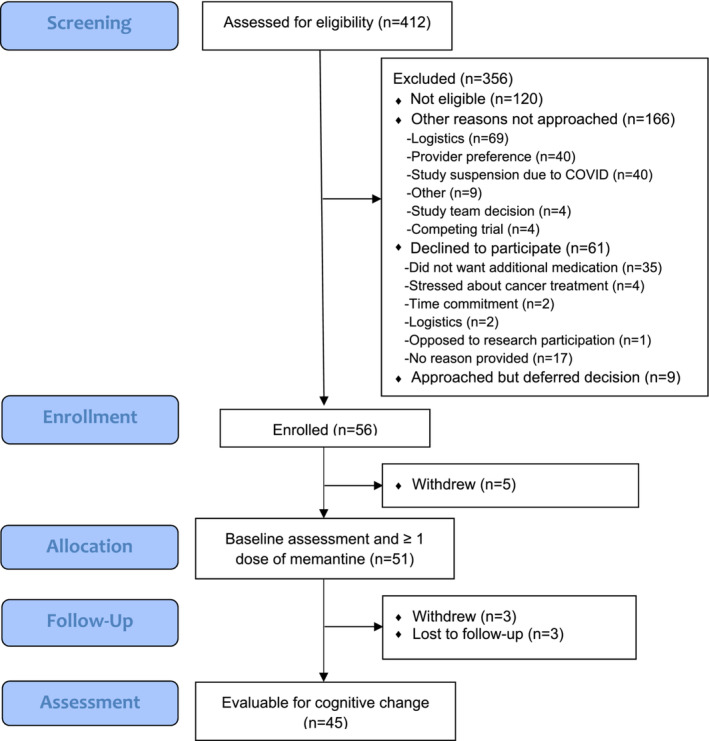
Modified CONSORT flow diagram for a single‐arm trial of memantine to prevent cognitive decline during chemotherapy for early‐stage breast cancer.

Of the 412 patients screened, 126 were eligible and approached, and 56 enrolled (recruitment rate: 44%; Figure 1). Reasons patients provided for declining participation included not wanting to start an additional medication (*n* = 35), stress about their cancer treatment (*n* = 4), time commitment concerns (*n* = 2), logistical barriers to completing required research activities (*n* = 2), and opposition to participating in research in general (*n* = 1). Fifty‐one participants initiated memantine. Forty‐five had at least one dose of memantine and completed both pre‐ and post‐assessments and, thus, were evaluable for cognitive change (retention rate: 80%). Thirty participants completed both assessments remotely, 11 completed one remotely and one in‐person, and four completed both in‐person.

On average, participants were scheduled for 22 weeks of memantine (SD 9.3). Thirty‐nine participants (76%) reported ≥90% adherence with scheduled doses, two participants (4%) reported 50%–89% adherence, and 10 participants (20%) reported <50% adherence. Independent of overall adherence, 12 participants (24%) discontinued memantine prior to the end of the study. Reasons for discontinuation included side effect concerns by the participant, provider, or study team regardless of formal attribution to memantine (*n* = 9), no specified reason (*n* = 2), and unrelated medical complications (*n* = 1). Six additional participants held memantine temporarily for a median duration of 3.4 weeks (range: 0.9–5.7).

### 3.3 Tolerability

AEs were generally low grade and not attributed to memantine (Table 2). Of the seven grade 3 AEs, only two (diarrhea and hypokalemia) had at least *possible* attribution to memantine. The most common AEs (regardless of attribution or severity) were fatigue, headache, constipation, diarrhea, and dizziness, which occurred in 20 (39%), 19 (37%), 14 (27%), 14 (27%), and 12 (24%) participants, respectively. Twenty‐nine participants (57%) had chemotherapy dose reduction. The most common reasons for dose reduction were peripheral neuropathy (*n* = 17), fatigue (*n* = 7), cytopenias (*n* = 4), elevated liver enzymes (*n* = 2), and diarrhea (*n* = 2).

**TABLE 2:** Adverse events

Attribution	NCI CTCAE grade			
	1	2	3	4
Unrelated	52	8	1	–
Unlikely	54	19	4	–
Possible	37	12	2	–
Probable	2	–	–	–
Definite	–	–	–	–

### 3.4 Acceptability

At the post‐assessment, 16.3% agreed or strongly agreed, 72.1% were unsure, and 11.6% disagreed or strongly disagreed that memantine had protected their cognition from becoming worse (Table 3). By comparison, 4.6% reported that memantine was a disruption to their lives and 7.0% expressed worries about having to take memantine.

**TABLE 3:** Responses to BMQ of memantine for cognition (*N* = 43)

Item	Agree or strongly agree (%)	Uncertain (%)	Disagree or strongly disagree (%)
My cognition, at present, depends on memantine	7.0	46.5	46.5
Having to take memantine worried me	7.0	7.0	86.0
My life would have been impossible without memantine	2.3	32.6	65.1
Without memantine I would have had problems with my cognition	13.9	55.8	30.3
I sometimes worried about long‐term effects of memantine	16.3	11.6	72.1
Memantine is a mystery to me	25.6	25.6	48.8
My future cognition will depend on memantine	2.3	30.2	67.4
Memantine disrupted my life	4.6	4.7	90.7
I sometimes worried about becoming too dependent on memantine	13.9	2.3	83.7
Memantine protected my cognition from becoming worse	16.3	72.1	11.6

### 3.5 Objective cognitive changes

Table 4 indicates the proportion of participants who improved, had no change, or declined across cognitive domains/measures. Most participants had no meaningful change. In the AWE domain, 22.2% improved, 64.4% had no change, and 13.3% declined. In the LM domain, 46.7% improved, 40.0% had no change, and 13.3% declined. In every measure except for the Animal naming test, more participants improved than declined. The DMS raw score improved by 3% (95% CI: −5% to +10%; range: −20% to +40%), which was not significantly different than the 5% raw score improvement in the historical control group of breast cancer patients who received chemotherapy without any intervention for CRCI^17^ (*p* = 0.55). Raw score changes across all cognitive measures are shown in Table S3. To explore impact of testing administration format, we compared baseline scores in those participants who completed the assessment in‐person (*n* = 20) versus those who completed the assessment remotely (*n* = 33). No meaningful differences were observed (Table S4).

**TABLE 4:** Cognitive changes from pre‐ to post‐assessment (*N* = 45)

Measure	Improved (%)	No change (%)	Declined (%)
AWE^a^	22.2	64.4	13.3
Digit span forward	15.6	68.9	15.6
Digit span backward	35.6	35.6	28.9
Controlled oral word association test	40.0	40.0	20.0
Animal naming	22.2	35.6	42.2
Delayed Matching to Sample	35.6	28.9	35.6
Rapid visual processing^b^	32.3	54.9	12.9
One Touch Stockings of Cambridge^b^	29.0	51.6	19.4
LM^c^	46.7	40.0	13.3
HVLT‐R total recall	48.9	35.6	15.6
HVLT‐R delayed recall	51.1	35.6	13.3
Global^d^	24.4	66.7	8.9

*Note*: Declined and improved defined by ≥0.5 SD change from pre‐ to post‐assessment; no change refers to <0.5 SD change from pre‐ to post‐assessment.

Abbreviations: AWE, attention, working memory, executive function; HVLT‐R, Hopkins Verbal Learning Test‐Revised; LM, learning and memory.

^a^ AWE: Composite of digit span forward and backward, Controlled Oral Word Association Test, Animal naming, Delayed Matching to Sample, Rapid Visual Processing, and One Touch Stockings of Cambridge.

^b^ Introduced at start of COVID‐19 pandemic. Completed in subset of *n* = 31.

^c^ LM: Composite of HVLT‐R total and delayed recall.

^d^ Global: Composite of all individual measures in the AWE and LM domains.

### 3.6 Patient‐reported cognitive changes

**FIGURE 2:**
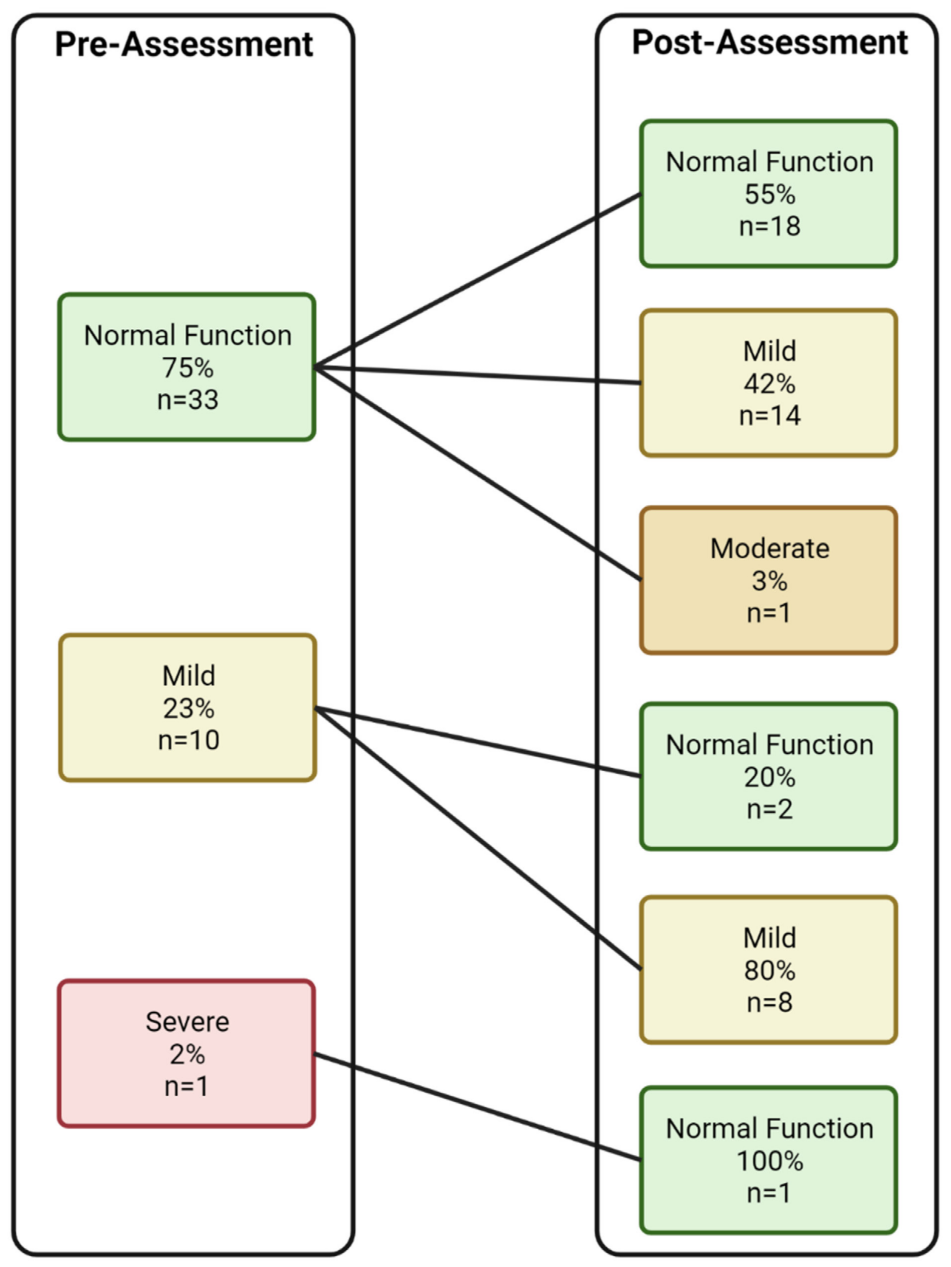
Changes in severity of self‐reported cognitive difficulties from pre‐ to post‐assessment (*N* = 44).

Overall, 66% (*n* = 29) demonstrated stability or improvement in patient‐reported cognition (Figure 2). Of the 33 participants with normal cognition at baseline, 18 remained within normal limits, 14 reported decline to mild cognitive difficulties, and one endorsed moderate post‐chemotherapy cognitive problems. Of those with mild difficulties at baseline, two improved to normal function and all others continued to report mild issues.

## 4 DISCUSSION

We have demonstrated the feasibility, tolerability, and acceptability of memantine in patients with early‐stage breast cancer undergoing chemotherapy. While we did not demonstrate efficacy in this single‐arm trial, a high proportion of participants demonstrated stable or improved cognition, supporting future study of memantine in an adequately powered, controlled trial.

There are limited data regarding the feasibility of pharmacological interventions for CRCI. However, in a trial of the stimulant methylphenidate administered during chemotherapy for breast cancer, researchers failed to reach target accrual.^26^ They reported challenges in participants' willingness to take another medication and about methylphenidate specifically. Two other studies involving methylphenidate for CRCI in the brain tumor population also closed prematurely due to slow accrual and/or high dropout.^27^, ^28^ By comparison, participants in our study reported few concerns about memantine and optimism about its potential to be helpful to them. Perhaps reflective of participants' concerns about CRCI and acceptability of memantine, rates of recruitment, retention, and adherence were all supportive of the feasibility of this intervention.

We are among the first to address a major research gap in interventions to prevent cognitive decline during chemotherapy. Specifically, most CRCI interventions have been tested years after completion of primary therapy. While there are many potential reasons for the lack of prevention studies, feasibility is likely among them. For example, a pilot study of the acetylcholinesterase inhibitor donepezil tested during radiation and chemotherapy in brain tumor patients closed prematurely.^29^ Yet, a pilot RCT of donepezil in breast cancer survivors who had completed adjuvant chemotherapy 1–5 years prior achieved target accrual over a 6‐month recruitment period, retained 76% of patients, and with minimal interruptions to treatment over the 24‐week protocol.^30^

The safety and tolerability of medications for CRCI, especially when administered during active treatment, is a critical issue. For example, approximately 15 years ago, several small studies of erythropoietin demonstrated mixed effects for CRCI,^6^ but there have been no additional studies after the FDA issued a warning about it increasing thromboembolic risk in cancer patients.^31^ Memantine has a strong track record of safety in Alzheimer's disease and other neurodegenerative disorders.^32^ More recently, a multi‐center study of its use during cranial radiation in patients with brain metastases to prevent cognitive decline showed that its toxicity profile was similar to placebo.^10^ While attribution of AEs of memantine is complicated by the fact that its most common side effects overlap with those secondary to chemotherapy, the prevalence of several of the most common AEs observed in our study, including fatigue, constipation, and diarrhea, were similar to that described in clinical practice.^33^

In this early phase study with focus on feasibility, we have contextualized the observed cognitive changes in our cohort to those in observational studies reporting cognitive change during breast cancer chemotherapy. There are several major challenges in comparing data to prior studies, including that most have been small studies, with heterogeneity in measures used to quantify CRCI, and variable approaches to account for the fact that scores on repeated cognitive testing are inflated by enhanced familiarity with the test (i.e., practice effects). Given these issues, we compared the change in our primary outcome (DMS % correct at 12 s delay) to that in the observational study by Janelsins and colleagues–the largest in breast cancer (*N* = 943) examining cognitive function at multiple timepoints relative to chemotherapy.^17^ There may be several potential reasons why we failed to demonstrate improvement over the historical control. First, the variance in the DMS in our sample was much larger than we had anticipated (and larger than that of any other measure in our study). Second, though several prior studies suggest that memantine targets working memory (the domain evaluated with the DMS) and related cognitive processes,^34^, ^35^, ^36^, ^37^ it is possible that another measure would have better captured the effects of memantine. Finally, it is also possible that memantine was not beneficial to patients in our study, but it is worth noting that our cohort improved on average on the digit span backward and Controlled Oral Word Association Test (two core CRCI measures) whereas studies by Janelsins et al. and others reported decline in these measures.^17^, ^38^, ^39^

It is also important to acknowledge that many patients with breast cancer do not experience cognitive decline during chemotherapy. In this study, we focused on the proportion of who experienced clinically meaningful changes in cognition. While prevalence estimates of the proportion of patients who decline in objectively measured cognition varies between ~15% and 50%,^17^, ^21^, ^38^, ^39^, ^40^, ^41^, ^42^, ^43^ the fact that we observed decline in ≤13% of participants across composite cognitive domains is encouraging. Further, on individual measures, most patients demonstrated no change from pre‐ to post‐assessment, and in eight of nine objective measures more patients demonstrated clinically meaningful improvement (beyond what would be expected from practice effects alone) than decline.

In our study, we objectively evaluated cognitive change using a traditional, neuropsychological battery of measures recommended by the International Cognition and Cancer Task Force^44^ as well as three computerized cognitive tests from the CANTAB battery. The decision to incorporate CANTAB was in response to a call for cognitive neuroscience measures in CRCI research to enhance sensitivity and disentangle deficits in specific cognitive processes not feasible with conventional neuropsychological testing. Additionally, results from the previously described observational study by Janelsins et al. support the sensitivity of the specific CANTAB measures used in our study.^17^, ^45^ In our planned RCT and other future work, we intend to expand the use of computerized cognitive neuroscience measures and extend beyond traditional accuracy or speed outcomes. For example, evaluating intraindividual variability in performance within and between tasks appears to be a more sensitive approach to identify subtle cognitive changes experienced during chemotherapy.^45^, ^46^, ^47^, ^48^, ^49^ In the future RCT, we also plan to facilitate interpretation of observed cognitive changes using reliable change indices^50^, ^51^ and enhance sensitivity to detect an effect using “gatekeeper” approaches,^52^, ^53^, ^54^ a method of condensing results from multiple cognitive tests into composite cognitive domain outcomes that has been demonstrated to increase reliability and precision of treatment estimates, improve power, and minimize Type I error, and, thus, is being increasingly utilized in CRCI and other populations.^22^, ^55^, ^56^, ^57^, ^58^

We also found promising early data as it relates to patient‐reported cognitive function. In Janelsins's cohort study, 45% of patients reported clinically significant decline.^59^ By comparison, in our study, 34% of participants reported clinically significant decline. While the patient‐report measure used in our study (PROMIS Short Form v2.0—Cognitive Function 8a) was not identical to what was used by Janelsins et al. (FACT‐Cog), the PROMIS Cognitive Function measure is almost entirely comprised of items from the FACT‐Cog. Additionally, the low levels of anxiety and depression in our sample and minimal change in these symptoms from pre‐ to post‐assessment suggest that the cognitive changes observed in our cohort were not simply reflective of changes in mental health.

### 4.1 Strengths and limitations

Strengths of our study include recruitment from multiple sites, high rate of data completion, and inclusion of both objective and patient‐reported cognitive outcomes. There are also notable limitations to acknowledge. Most significantly, the use of a historical control limited our ability to make direct comparisons to evaluate efficacy, and a follow‐up randomized trial is planned for this purpose. Regarding generalizability, participants who enrolled in our study may have had more concerns about their cognition entering treatment and/or were more optimistic about memantine's potential to benefit them, which may have led to bias, especially in patient‐reported symptoms. Participants also had high levels of education and were about 5 years younger than the average age of patients diagnosed with breast cancer.^60^ Thus, our sample may have had greater cognitive reserve and more resilience to the cognitive impact of their cancer and its treatment. Otherwise, patient and treatment characteristics were largely reflective of clinical populations in the United States Finally, conducting this study during the COVID‐19 pandemic required mid‐study protocol modifications (e.g., mixed in‐person and remote cognitive assessments). However, there is research supporting the equivalency of in‐person and remote cognitive testing and the use of teleneuropsychology during the pandemic has been accepted as a reliable and valid alternative to in‐person assessment.^61^, ^62^, ^63^, ^64^ To our knowledge, there have not yet been published studies examining the reliability and validity of teleneuropsychology in breast cancer, but its feasibility and acceptability have been demonstrated in other cancer populations.^65^

### 4.2 Conclusions and future directions

This is one of the first studies to demonstrate that a medication for prevention of cognitive decline during chemotherapy is feasible. As indicated above, we plan to extend this work to next conduct a RCT of memantine in this patient population and treatment setting. Because CRCI is a heterogeneous phenomenon with some patients experiencing substantial decline and others being unaffected, future research aimed at recognizing who is most at risk for cognitive decline during chemotherapy and who may experience most benefit from specific interventions is needed.

## AUTHOR CONTRIBUTIONS

**Zev M. Nakamura:** Conceptualization (lead); data curation (lead); formal analysis (supporting); funding acquisition (lead); investigation (lead); methodology (lead); project administration (lead); supervision (lead); validation (lead); visualization (lead); writing – original draft (lead); writing – review and editing (lead). **Allison M. Deal:** Conceptualization (supporting); data curation (supporting); formal analysis (lead); methodology (supporting); visualization (supporting); writing – review and editing (supporting). **Eliza M. Park:** Conceptualization (supporting); methodology (supporting); project administration (supporting); supervision (supporting); writing – original draft (supporting); writing – review and editing (supporting). **Kate E. Stanton:** Data curation (supporting); investigation (supporting); resources (supporting); software (supporting); writing – review and editing (supporting). **Yesy E. Lopez:** Data curation (supporting); investigation (supporting); resources (supporting); software (supporting); writing – review and editing (supporting). **Laura J. Quillen:** Data curation (supporting); investigation (supporting); resources (supporting); software (supporting); writing – review and editing (supporting). **Erin O'Hare Kelly:** Investigation (supporting); resources (supporting); writing – review and editing (supporting). **Hillary M. Heiling:** Data curation (supporting); formal analysis (supporting); visualization (supporting); writing – review and editing (supporting). **Kirsten A. Nyrop:** Project administration (supporting); supervision (supporting); writing – review and editing (supporting). **Emily M. Ray:** Resources (supporting); writing – review and editing (supporting). **E. Claire Dees:** Resources (supporting); writing – review and editing (supporting). **Katherine E. Reeder‐Hayes:** Resources (supporting); writing – review and editing (supporting). **Trevor A. Jolly:** Resources (supporting); writing – review and editing (supporting). **Lisa A. Carey:** Resources (supporting); writing – review and editing (supporting). **Yara Abdou:** Resources (supporting); writing – review and editing (supporting). **Oludamilola A. Olajide:** Resources (supporting); writing – review and editing (supporting). **Julia K. Rauch:** Resources (supporting); writing – review and editing (supporting). **Ranjit Joseph:** Resources (supporting); writing – review and editing (supporting). **Anureet Copeland:** Resources (supporting); writing – review and editing (supporting). **Megan A. McNamara:** Project administration (supporting); resources (supporting); writing – review and editing (supporting). **Tim A. Ahles:** Conceptualization (supporting); methodology (supporting); project administration (supporting); supervision (supporting); writing – original draft (supporting); writing – review and editing (supporting). **Hyman B. Muss:** Conceptualization (supporting); funding acquisition (supporting); methodology (supporting); project administration (lead); supervision (supporting); writing – original draft (supporting); writing – review and editing (supporting).

## FUNDING INFORMATION

This study was supported by the National Institutes of Health (NIH; Grant # 5K12HD001441, Scholar: ZMN; P30CA008748, TAA; 5R01CA218496, TAA; 5K07CA218167, EMP; 5P50CA058223; 5P30CA016086), Breast Cancer Research Foundation (New York, NY; HBM), Kay Yow Foundation (Raleigh, NC; HBM), and the University Cancer Research Fund. The content is solely the responsibility of the authors and does not represent the official views of the NIH.

## CONFLICT OF INTEREST

Emily Ray receives research funding from OptumHealth and Pfizer. E. Claire Dees consults for Sanofi and receives research funding from Novartis, Genentech/Roche, Pfizer, Merck, H3 Biomedicine, and Meryx Pharmaceuticals. Katherine Reeder‐Hayes receives research funding from Pfizer. Lisa Carey receives research funding from Syndax, Novartis, NanoString Technologies, Abbvie, Seattle Genetics, and Veracyte. Yara Abdou consults for Exact Sciences.

## Supporting information


Tables S1‐S4

## Data Availability

The data that support the findings of this study are available from the corresponding author upon reasonable request.
