# Supplementary material for: A phase II single‐arm trial of memantine for prevention of cognitive decline during chemotherapy in patients with early breast cancer: Feasibility, tolerability, acceptability, and preliminary effects
Source: Cancer Med. 2023 Jan 16;12(7):8172–83. doi: 10.1002/cam4.5619 (PMC10134315; doi:10.1002/cam4.5619)
Supplement: Supplementary file 1 — Tables S1‐S4 [file CAM4-12-8172-s001.docx]

**Supplemental Table 1. Criteria for Attribution to Memantine**

| **Attribution** | **Criteria** | **# Needed** |
| --- | --- | --- |
| Unrelated | No extraneous causes | --- |
| Unlikely | 1.) Did not have a temporal relationship  2.) Could not readily have been produced by the participant’s clinical state  3.) Could have been due to environmental or other interventions  4.) Does not follow a known pattern of response  5.) Does not reappear or worsen with reintroduction | ≥ 2 |
| Possible | 1.) Reasonable temporal relationship  2.) Could not readily have been produced by participant’s clinical state  3.) Could not have been due to environmental or other interventions  4.) Follows a known pattern of response | ≥ 2 |
| Probable | 1.) Reasonable temporal relationship  2.) Could not readily have been produced by the participant’s clinical state or environmental or other interventions  3.) Follows a known response  4.) Disappears or decreases with reduction in dose or cessation of intervention | 3 |
| Definite |  | 4 |

**Supplemental Table 2. Objective Neuropsychological Outcome Measures**

| **Measure** | **Type** | **Domain(s)** |
| --- | --- | --- |
| AWE* |  | Attention, Working Memory, Executive Function |
| Delayed Matching to Sample | Computer-based | Visual Working Memory |
| Rapid Visual Processing | Computer-based | Processing Speed, Attention |
| One Touch Stockings of Cambridge | Computer-based | Executive Function |
| Digit Span Forwards | Paper-based | Attention |
| Digit Span Backwards | Paper-based | Working Memory |
| Controlled Oral Word Association Test | Paper-based | Phonemic Fluency, Executive Function |
| Animal Naming | Paper-based | Semantic Fluency, Executive Function |
| LM** |  | Learning and Memory |
| HVLT-R Total Recall | Paper-based | Verbal Learning and Memory |
| HVLT-R Delayed Recall | Paper-based | Verbal Learning and Memory |

Abbreviations: AWE – attention, working memory, executive function; LM – learning and memory; HVLT-R – Hopkins Verbal Learning Test-Revised

*AWE: Composite of Digit Span Forwards and Backwards, Controlled Oral Word Association Test, Animal Naming, Delayed Matching to Sample, Rapid Visual Processing, and One Touch Stockings of Cambridge

**LM: Composite of HVLT-R Total and Delayed Recall

**Supplemental Table 3. Cognitive Measure Scores at Pre- and Post-Assessment (N=45)**

|  | **Mean (SD)** | | |
| --- | --- | --- | --- |
| **Measure** | **Pre** | **Post** | **Δ Pre - Post*** |
| AWE | 0.18 (0.61) | 0.29 (0.68) | 0.11 (0.48) |
| Digit Span Forwards | 10.33 (2.09) | 10.53 (2.36) | 0.20 (1.84) |
| Digit Span Backwards | 9.44 (2.24) | 9.91 (2.90) | 0.47 (3.01) |
| Controlled Oral Word Association Test | 41.27 (11.97) | 42.93 (11.50) | 1.67 (8.78) |
| Animal Naming | 22.96 (5.83) | 21.82 (5.28) | -1.13 (4.42) |
| Delayed Matching to Sample | 81.78 (20.37) | 84.44 (17.00) | 2.67 (26.15) |
| Rapid Visual Processing** | 0.89 (0.06) | 0.89 (0.08) | 0.01 (0.05) |
| One Touch Stockings of Cambridge** | 11.19 (2.12) | 11.45 (2.35) | 0.26 (2.25) |
| LM | -0.14 (1.08) | 0.23 (1.01) | 0.37 (0.70) |
| HVLT-R Total Recall | 26.49 (5.16) | 28.00 (4.85) | 1.51 (4.06) |
| HVLT-R Delayed Recall | 9.29 (2.11) | 10.09 (2.02) | 0.80 (1.52) |
| Global | 0.11 (0.61) | 0.29 (0.67) | 0.17 (0.42) |
| PROMIS Short Form v2.0 – Cognitive Function 8a *** | 49.29 (8.53) | 47.57 (8.47) | -0.19 (0.93) |

Abbreviations: AWE – attention, working memory, executive function; LM – learning and memory; HVLT-R – Hopkins Verbal Learning Test-Revised; PROMIS – Patient-reported Outcome Measurement Information System

*Notes:* AWE, LM, and Global are Z-scores of individual measures standardized based on available age-, sex-, education-, and race/ethnicity-matched normative data. PROMIS Cognitive Function are T-Scores standardized based on population data. All other measures are raw measure scores without adjustment.

*positive values indicate better post- compared to pre-assessment score; ^**^n=31; ^***^n=44

**Supplemental Table 4. Comparison of In-Person and Virtual Pre-Intervention Assessment Performance (N=53)**

|  | **Mean (SD)** | |  |
| --- | --- | --- | --- |
| **Measure** | **In-Person (n=20)** | **Virtual (n=33)** | **p** |
| AWE | 0.18 (0.71) | 0.21 (0.55) | 0.89 |
| Digit Span Forwards | 9.85 (2.32) | 10.73 (1.91) | 0.14 |
| Digit Span Backwards | 9.05 (2.70) | 9.85 (2.05) | 0.23 |
| Controlled Oral Word Association Test | 41.50 (13.81) | 39.67 (11.06) | 0.57 |
| Animal Naming | 21.45 (4.76) | 22.88 (6.37) | 0.39 |
| Delayed Matching to Sample | 84.00 (17.89) | 83.03 (20.69) | 0.86 |
| Rapid Visual Processing* | 0.82 (0.01) | 0.89 (0.06) | 0.13 |
| One Touch Stockings of Cambridge* | 7.00 (1.41) | 11.45 (1.89) | **0.003** |
| LM | 0.11 (0.82) | -0.27 (1.21) | 0.21 |
| HVLT-R Total Recall | 27.15 (4.68) | 26.21 (5.69) | 0.54 |
| HVLT-R Delayed Recall | 9.75 (1.86) | 9.12 (2.27) | 0.30 |
| Global | 0.16 (0.69) | 0.10 (0.57) | 0.72 |
| PROMIS Short Form v2.0 – Cognitive Function 8a ** | 50.58 (10.79) | 48.51 (7.04) | 0.46 |

Abbreviations: AWE – attention, working memory, executive function; LM – learning and memory; HVLT-R – Hopkins Verbal Learning Test-Revised; PROMIS – Patient-reported Outcome Measurement Information System

*Notes:* AWE, LM, and Global are Z-scores of individual measures standardized based on available age-, sex-, education-, and race/ethnicity-matched normative data. PROMIS Cognitive Function are T-Scores standardized based on population data. All other measures are raw measure scores without adjustment.

*n=35 (2 in-person, 33 virtual)

**n=51 (19 in-person, 32 virtual)
